# The efficacy and safety of third-generation antiseizure medications and non-invasive brain stimulation to treat refractory epilepsy: a systematic review and network meta-analysis study

**DOI:** 10.3389/fneur.2023.1307296

**Published:** 2024-01-09

**Authors:** Yang Yang, Yafei Shangguan, Xiaoming Wang, Ruihong Liu, Ziyi Shen, Ming Tang, Guohui Jiang

**Affiliations:** ^1^Department of Neurology, Affiliated Hospital of North Sichuan Medical College, Institute of Neurological Diseases, North Sichuan Medical College, Nanchong, China; ^2^Department of Neurology, The First People’s Hospital of Guiyang, Guiyang, China

**Keywords:** third-generation antiseizure medications, epilepsy, non-invasive brain stimulation, network meta-analysis, refractory epilepsy

## Abstract

**Background:**

The new antiseizure medications (ASMs) and non-invasive brain stimulation (NIBS) are controversial in controlling seizures. So, this network meta-analysis aimed to evaluate the efficacy and safety of five third-generation ASMs and two NIBS therapies for the treatment of refractory epilepsy.

**Methods:**

We searched PubMed, EMBASE, Cochrane Library and Web of Science databases. Brivaracetam (BRV), cenobamate (CNB), eslicarbazepine acetate (ESL), lacosamide (LCM), perampanel (PER), repetitive transcranial magnetic stimulation (rTMS), and transcranial direct current stimulation (tDCS) were selected as additional treatments for refractory epilepsy in randomized controlled studies and other cohort studies. Randomized, double-blind, placebo-controlled, add-on studies that evaluated the efficacy or safety of medication and non-invasive brain stimulation and included patients with seizures were uncontrolled by one or more concomitant ASMs were identified. A random effects model was used to incorporate possible heterogeneity. The primary outcome was the change in seizure frequency from baseline, and secondary outcomes included the proportion of patients with ≥50% reduction in seizure frequency, and the rate of treatment-emergent adverse events.

**Results:**

Forty-five studies were analyzed. The five ASMs and two NIBS decreased seizure frequency from baseline compared with placebo. The 50% responder rates of the five antiseizure drugs were significantly higher than that of placebo, and the ASMs were associated with fewer adverse events than placebo (*p* < 0.05). The surface under the cumulative ranking analysis revealed that ESL was most effective in decreasing the seizure frequency from baseline, whereas CNB provided the best 50% responder rate. BRV was the best tolerated. No significant publication bias was identified for each outcome index.

**Conclusion:**

The five third-generation ASMs were more effective in controlling seizures than placebo, among which CNB, ESL, and LCM were most effective, and BRV exhibited better safety. Although rTMS and tDCS did not reduce seizure frequency as effectively as the five drugs, their safety was confirmed.

**Systematic review registration:**

PROSPERO, https://www.crd.york.ac.uk/prospero/ (CRD42023441097).

## Introduction

Epilepsy is a long-term neurological condition marked by repeated seizures, and it frequently correlates with irregularities in cognitive function, mental well-being, and social adaptability (1). Epilepsy impacts a minimum of 1.2% of the global population. Most patients with epilepsy can be seizure-free after taking antiseizure medications (ASMs); however, at least one-third of patients develop resistance to ASMs and develop refractory epilepsy, which poses a serious public health problem with high economic costs (2). According to the definition of drug-resistant epilepsy proposed by the International League Against Epilepsy (ILAE) in 2010, the right choice and use of two ASMs cannot achieve sustained seizure freedom which can be considered as drug-resistant epilepsy or refractory epilepsy (3). Refractory epilepsy does not mean that any drug treatment cannot control seizures, but as the course of uncontrollable seizures is prolonged, the responsiveness of new treatments may also be reduced (4). Therefore, it is particularly important to determine the effective treatments in time.

There are several hypotheses about the mechanisms of drug resistance in epilepsy, such as transporter hypothesis, target hypothesis, neural network change hypothesis, neuroinflammation hypothesis, and so on (5, 6). Different patients with refractory epilepsy have personalized resistance mechanisms, and there may be one or more resistance mechanisms at the same time (7). The mechanisms of drug-resistant epilepsy are complex and vary from person to person, so it is necessary to provide individualized antiseizure therapies including drug and non-drug treatments.

Currently, the main treatment for epilepsy is drug therapy. A variety of ASMs are available, among which new ASMs have fewer adverse reactions, while effectively controlling epileptic seizures (8). In the past decade, five “third-generation” ASMs, namely, brivaracetam (BRV), cenobamate (CNB), eslicarbazepine acetate (ESL), lacosamide (LCM) and perampanel (PER) have been approved as adjunctive therapies for adult patients with epilepsy (9). However, the above drugs can only relieve the seizure to a certain extent, but do not reverse the disease. Each type of new ASMs have their own characteristics and scope of application. When selecting ASMs for treatment, physicians must carefully consider and compare the efficacy and safety profiles of the medications in order to provide useful clinical guidance for managing patients with epilepsy.

Surgical intervention is another treatment option for intractable epilepsy. However, surgery is not suitable for all patients with refractory epilepsy due to its high risk, inability to completely control seizures after surgery, and even some sequelae and neurological dysfunction (10). Alternative treatments must be developed. Non-invasive brain stimulation (NIBS) modulates brain excitability and encompasses a range of techniques, including repetitive transcranial magnetic stimulation (rTMS) and transcranial direct current stimulation (tDCS) (11). In recent years, a substantial body of evidence has emerged regarding the efficacy of non-invasive brain stimulation (NIBS), particularly repetitive transcranial magnetic stimulation (rTMS) and transcranial direct current stimulation (tDCS). NIBS is painless and non-invasive, and several open-label studies have suggested that rTMS and tDCS exhibit significant antiseizure effect (12–15). tDCS decreases cortical excitability through cathode stimulation, and increases cortical excitability through anode stimulation (16). High-frequency rTMS enhances cortical excitability and may increase the risk of seizures, whereas low-frequency rTMS reduces cortical excitability (17). Some studies have shown that rTMS and tDCS only provide short-term reduction of seizures in patients, whereas other studies demonstrated no significant difference in efficacy compared to placebo treatment (18, 19). NIBS therapies are generally safe and do not cause significant side effects or complications. The treatment usually does not cause pain or discomfort, and patients can be treated in a comfortable environment (20). However, there is currently insufficient data to draw definitive conclusions regarding the antiseizure potential of rTMS or tDCS, thus requiring further research.

Network meta-analyses enable direct and indirect comparisons. Therefore, this systematic review and network meta-analysis aimed to compare the efficacy and safety of different interventions in the treatment of refractory epilepsy through network meta-analysis, in order to identify the interventions with the best clinical outcomes and provide guidance for clinical decision-making. Using placebo as a control, we focused on the efficacy of third-generation ASMs and NIBS as additional treatments to control seizures in patients with refractory epilepsy, as well as the incidence of TEAE during treatment.

## Methods

We adhered to the guidelines of the preferred reporting items for systematic reviews and meta-analyses (PRISMA) to conduct this study (21). This review is based on previous research and does not include new research in human participants. This systematic review and network meta-analysis has been registered on PROSPERO (CRD 42023441097).

### Literature retrieval strategy

PubMed, EMBASE, Cochrane Library and Web of Science databases were searched from inception to April 2023. The language of publication is restricted to English. A random effect model was used to incorporate possible heterogeneity. The following search terms were used: [“Seizures” (Mesh) OR “Epilepsy” (Mesh)] AND [“Transcranial Magnetic Stimulation” (Mesh) OR “Transcranial Direct Current Stimulation” (Mesh)] OR [“repetitive transcranial magnetic stimulation” (Title/Abstract) OR “TMS” (Title/Abstract) OR “rTMS” (Title/Abstract) OR “tDCS” (Title/Abstract) OR “brain polarization” (Title/Abstract) OR “galvanic stimulation” (Title/Abstract) OR “eslicarbazepine acetate” (Title/Abstract) OR “perampanel” (Title/Abstract) OR “lacosamide” (Title/Abstract) OR “brivaracetam” (Title/Abstract) OR “cenobamate” (Title/Abstract)].

### Eligibility criteria

Randomized, double-blind, placebo-controlled, add-on studies that evaluated the efficacy and safety of medication and non-invasive brain stimulation in the treatment of patients with seizures that was uncontrolled by one or more concomitant ASMs. Concomitant ASMs had been kept stable before trial entry and throughout the treatment periods. The participant agreed to keep the ASMs unchanged throughout the whole study. We included high-quality clinical trials in English, including RCTs and cohort studies with Newcastle–Ottawa scale (NOS) quality scores ≥5.

The exclusion criteria were as follows: (1) reports of reviews or meetings; (2) studies in which the outcome measures did not describe seizure frequency; (3) concomitant ASMs had been kept unstable before trial entry and throughout the treatment periods; (4) studies with no placebo control; and (5) cohort studies with Newcastle–Ottawa scale (NOS) quality scores <5 (22, 23) and RCTs considered to be low quality after Cochrane risk of bias assessment.

### Outcome measures

The primary outcome was the change in seizure frequency from baseline (seizure response) after treatment. The secondary outcomes included the proportion of patients with ≥50% reduction in seizure frequency (defined as responders), and the rates of treatment-emergent adverse events (TEAEs).

### Study selection, data extraction, and evaluation of the quality of the included studies

We conducted an extensive literature search to collect research studies that fit our research objectives. The databases we used included PubMed, EMBASE, Cochrane Library and Web of Science databases. During the screening process, three independent researchers conducted an initial screening of the literature, evaluating it based on the relevance of the title and abstract. Subsequently, the full text of the literature meeting the screening criteria was read to finalize the articles included in the study. To extract the data, we designed a standardized data extraction table, as shown in Supplementary Table S1, which was used by three independent investigators to extract data from each included study. If disagreements arose, they were resolved through discussions with other researchers in our team until a consensus was reached. The extracted data included the title, year of publication, author(s), number of participants, study design, intervention, and outcome measures.

The data were synthesized and analyzed using the RevMan 5.4 software to assess the risk of bias. The risk of bias in the included RCTs was assessed according to the recommendations of the Cochrane Collaboration (24). The NOS quality scores was used to evaluate the quality of the cohort studies.

### Statistical analysis

RevMan 5.4.1 and Stata 15.1 were used to analyze and process the data. Measurement data are expressed as the mean difference (MD), and the odds ratio (OR) was adopted for numerical data. We ranked the interventions for each outcome by calculating the surface under the cumulative ranking curve (SUCRA) probabilities.

SUCRA is a percentage between 0 and 100 that represents the relative position of each treatment measure in all possible rankings (25). SUCRA values can provide a simple and intuitive way to help decision makers make rational choices between multiple treatment options. Publication bias in the included trials was assessed by generating a funnel plot using the Stata 15 software. Statistical significance was set at *p* < 0.05.

## Results

### Literature search results and quality evaluation

**Figure 1:**
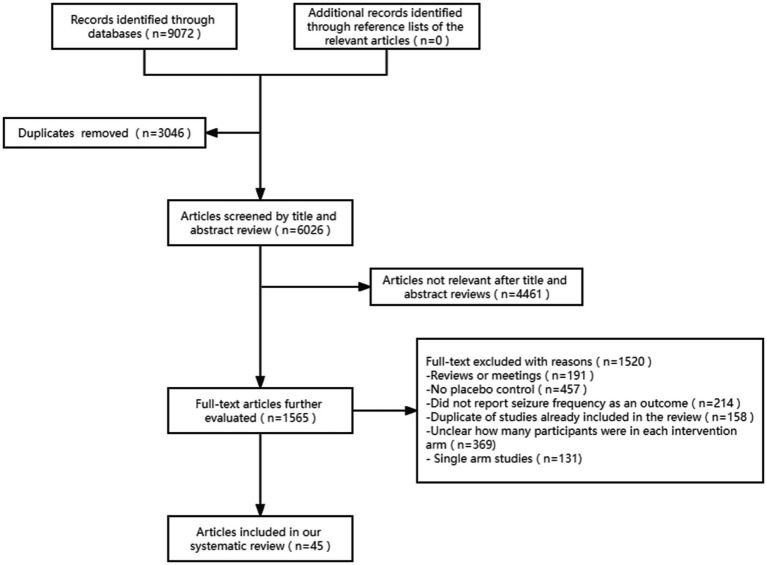
Flow diagram of the literature screening process.

The literature search using the above English databases retrieved 9,072 studies. After review, 9,027 of these studies were excluded because they did not meet the inclusion criteria, and 45 studies were included in the network meta-analysis. The article retrieval process is illustrated in Figure 1.

**Figure 2:**
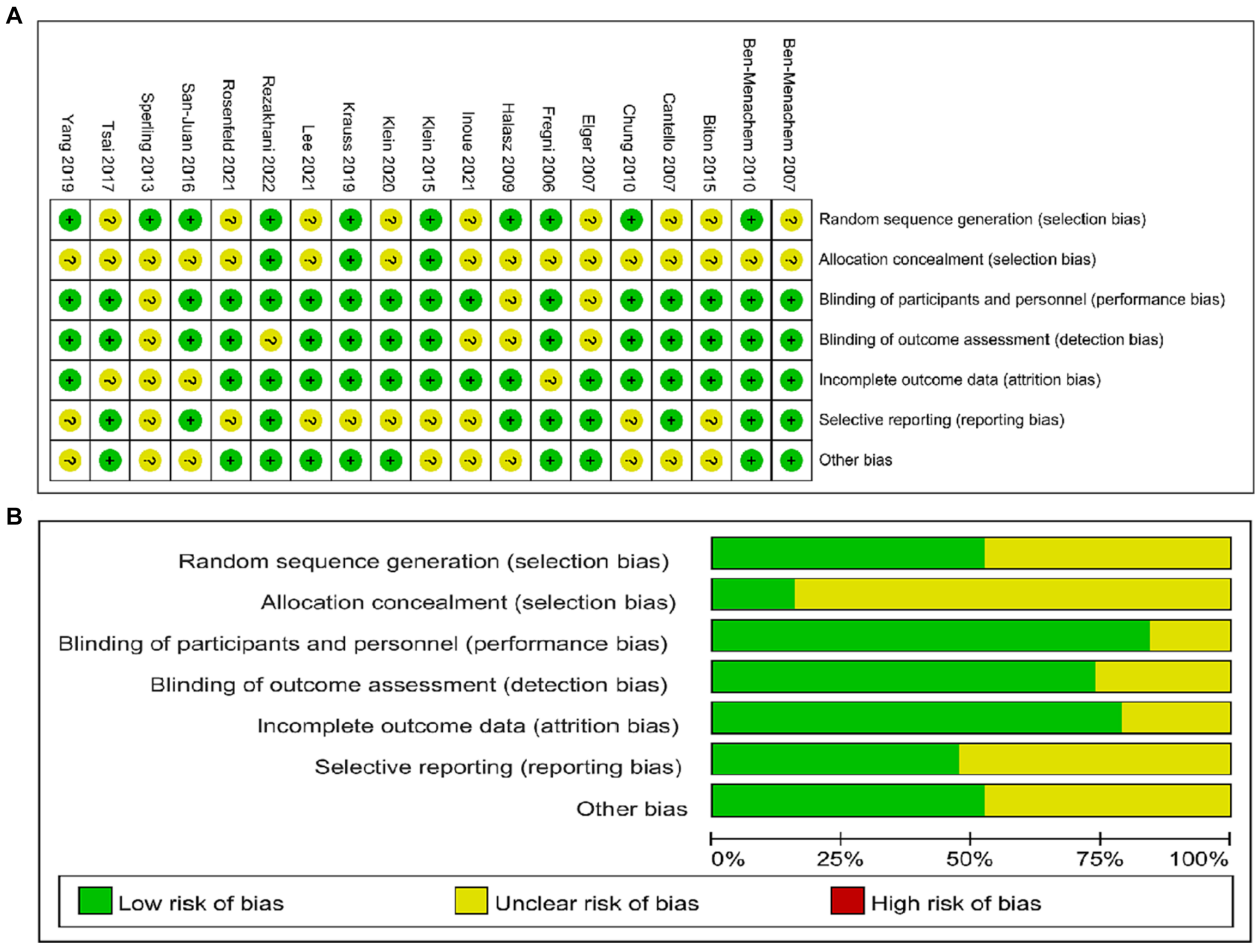
Evaluation of quality of included studies. **(A)** Review of authors’ judgements for each risk of bias item. **(B)** Review of authors’ judgements for bias item for each study.

The final studies included 28,819 patients (tDCS: 79, rTMS: 47, BRV: 4350, CNB: 674, ESL: 2347, LCM: 3063, PER: 9120, and placebo: 9139). Additional details of the included studies are provided in Supplementary Table S1. The results of the Cochrane risk of bias assessment of the 19 included RCTs using RevMan5.4 software are shown in Figure 2. The NOS scores are presented in Supplementary Table S1 and indicate that the included cohort studies were of high quality.

### Presentation of network structure

**Figure 3:**
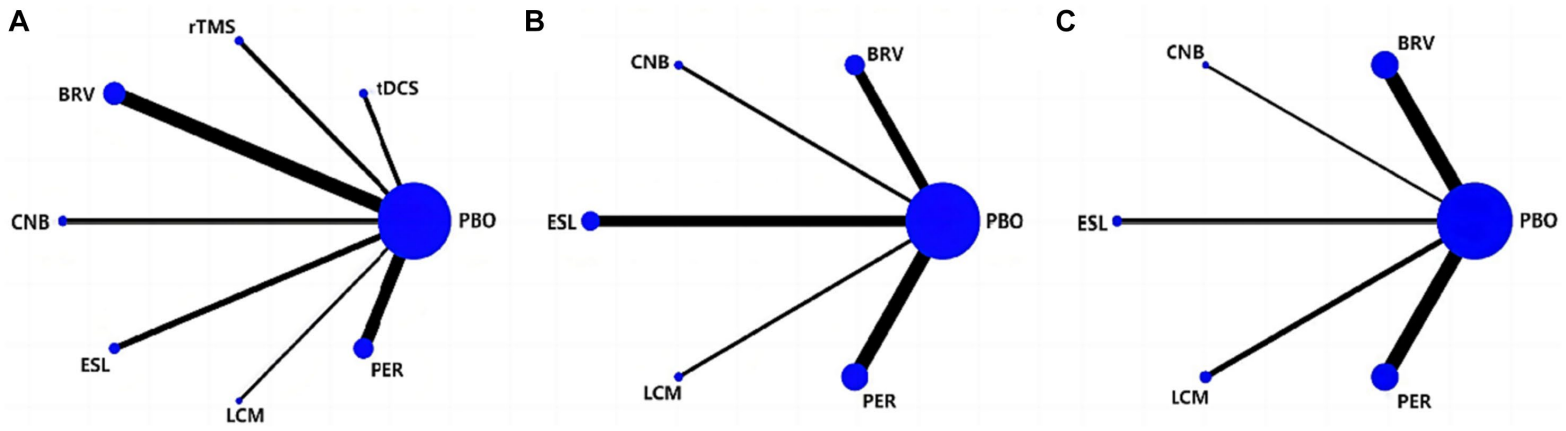
Network of eligible treatment comparisons for seizure frequency reduction from baseline **(A)**, 50% responder rate **(B)**, and TEAEs **(C)**. TEAE, treatment-emergent adverse event; PBO, placebo; BRV, brivaracetam; CNB, cenobamate; ESL, eslicarbazepine acetate; LCM, lacosamide; PER, perampanel; rTMS, repetitive transcranial magnetic stimulation; tDCS, transcranial direct current stimulation.

Figure 3A depicts the network geometry of the interactions based on therapeutic evaluations of seizure frequency reduction from baseline. Figure 3B illustrates the network geometry of the interactions based on ≥50% responder rate, and Figure 3C details the network geometry of the interactions based on the rate of TEAEs. The size of the node represents the number of trials per intervention and control group; the larger the node size, the more trials the corresponding node contains. The thickness of the line between the corresponding nodes would indicate the number of comparisons between the two interventions. However, there was no direct comparison between any two interventions, and they were both compared with the placebo group. Hence, this network geometry can be used for direct and indirect evaluation comparisons.

### Therapeutic evaluation of seizure frequency reduction from baseline

**Figure 4:**
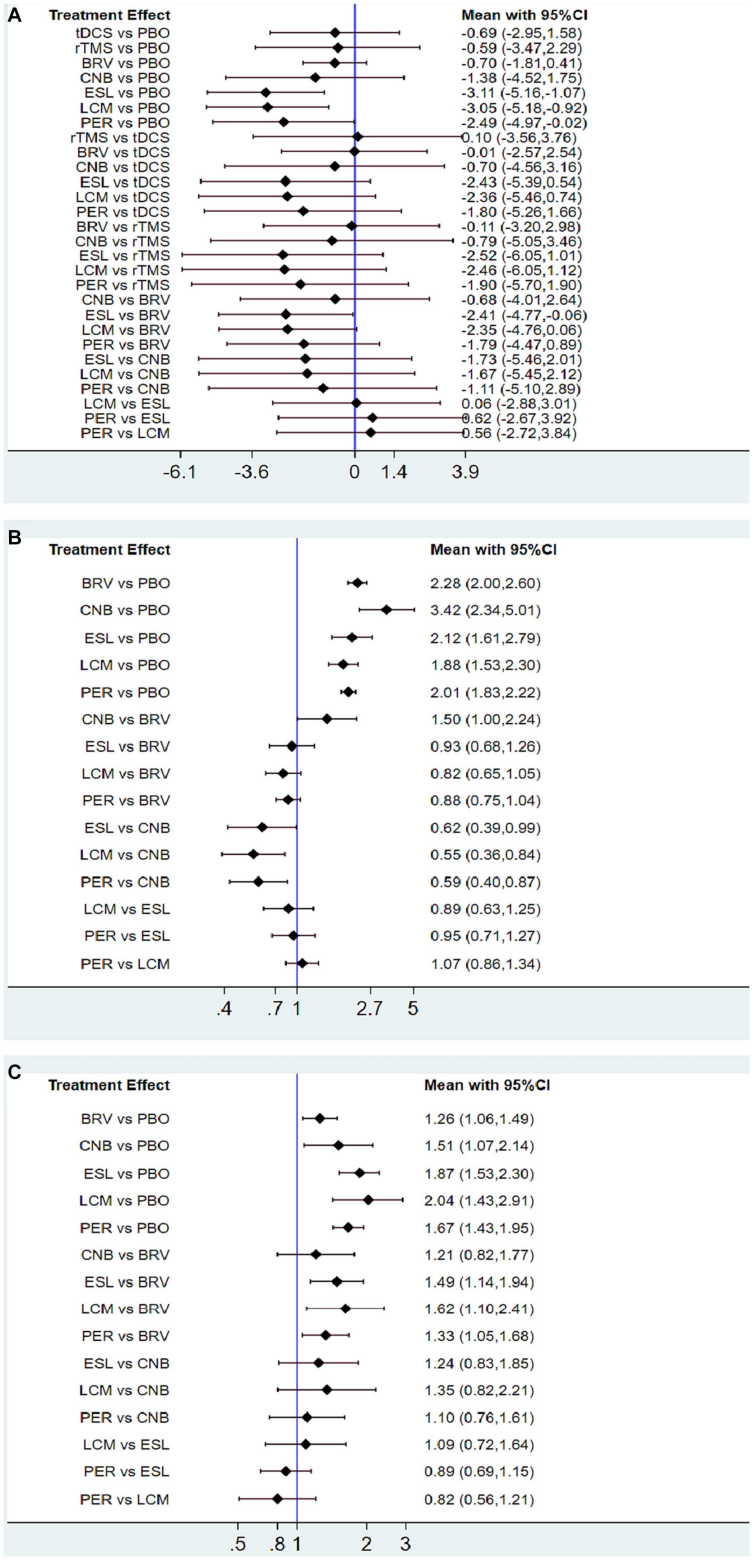
Interval plot for intervention effect size on seizure frequency reduction from baseline **(A)**, 50% responder rate **(B)**, and TEAEs **(C)**. TEAE, treatment-emergent adverse event; PBO, placebo; BRV, brivaracetam; CNB, cenobamate; ESL, eslicarbazepine acetate; LCM, lacosamide; PER, perampanel; rTMS, repetitive transcranial magnetic stimulation; tDCS, transcranial direct current stimulation.

**Figure 5:**
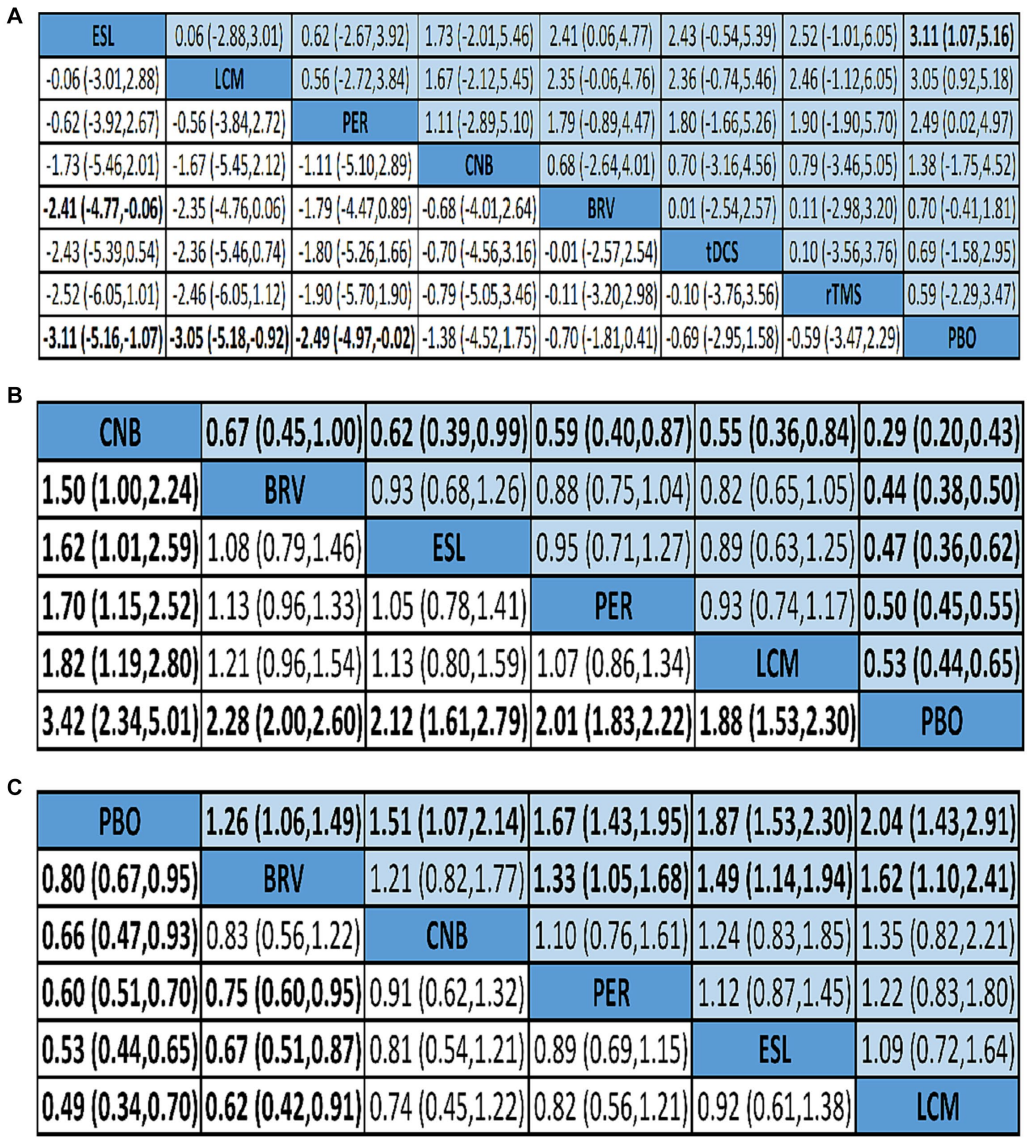
Network meta-analysis league map of outcomes: seizure frequency reduction from baseline **(A)**, 50% responder rate **(B)**, and TEAEs **(C)**. TEAE, treatment-emergent adverse event; PBO, placebo; BRV, brivaracetam; CNB, cenobamate; ESL, eslicarbazepine acetate; LCM, lacosamide; PER, perampanel; rTMS, repetitive transcranial magnetic stimulation; tDCS, transcranial direct current stimulation.

As shown in Figures 4A, 5A, the ESL group that compared with placebo demonstrated the most favorable treatment effect [MD: −3.11 (95% CI −5.16 to −1.07)], indicating the greatest reduction in seizure frequency from baseline (high-quality evidence, 2,347 participants). For the non-drug therapies, the rTMS group exhibited a worse effect than the tDCS group [0.10 (95% CI −3.56 to 3.76)]. However, this difference was not statistically significant.

**Figure 6:**
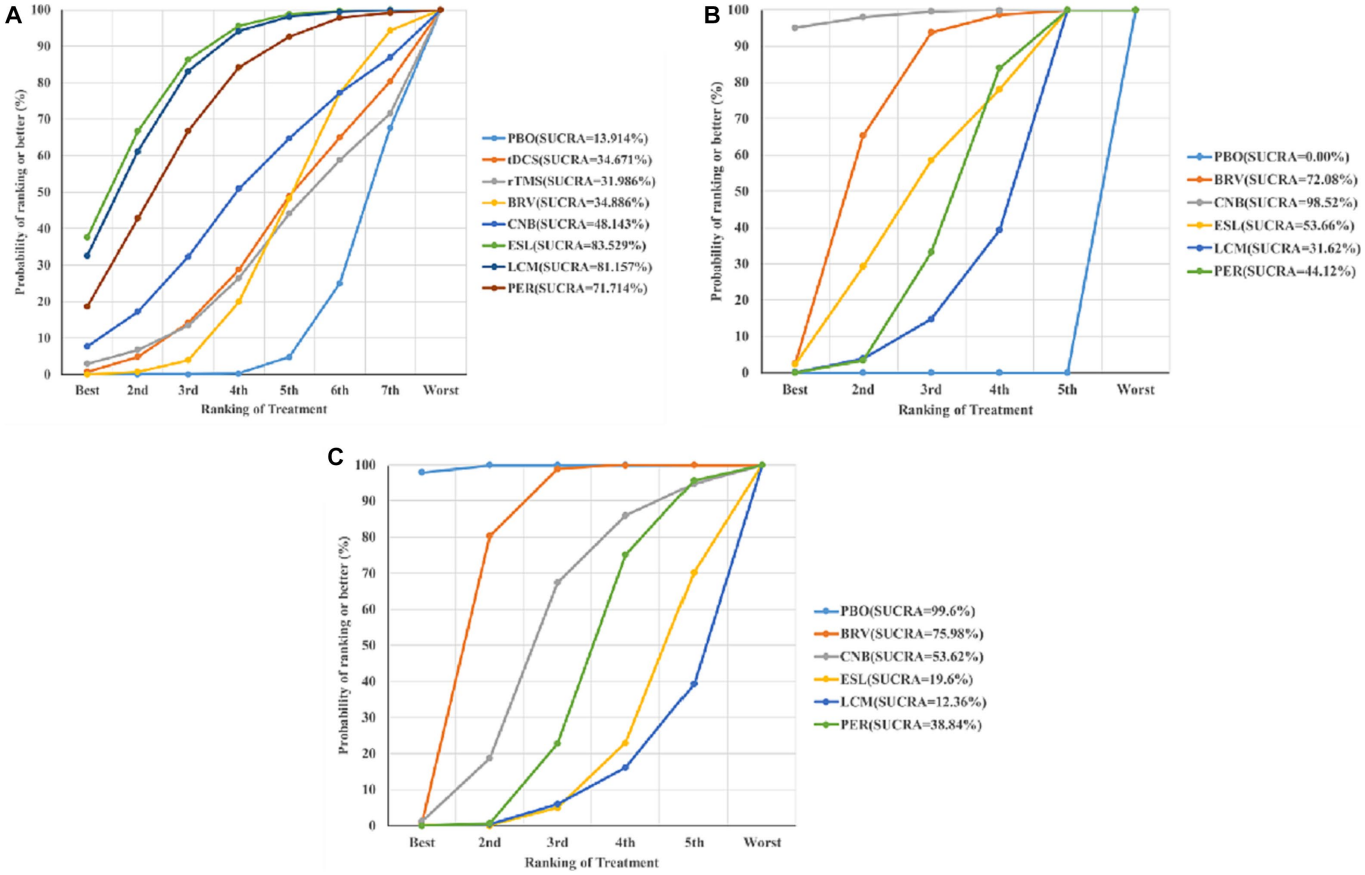
Ranking of treatment strategies according to the SUCRA probabilities: seizure frequency reduction from baseline **(A)**, 50% responder rate **(B)**, and TEAEs **(C)**. SUCRA, surface under the cumulative ranking curve; TEAE, treatment-emergent adverse event; PBO, placebo; BRV, brivaracetam; CNB, cenobamate; ESL, eslicarbazepine acetate; LCM, lacosamide; PER, perampanel; rTMS, repetitive transcranial magnetic stimulation; tDCS, transcranial direct current stimulation.

The effects of all treatments were ranked using SUCRA probabilities (Figure 6A), and the ESL achieved the highest probability (SUCRA 83.529%) of being the best treatment to reduce seizure frequency from baseline, followed by LCM (SUCRA 81.157%), PER (SUCRA 71.714%), and placebo (SUCRA 13.914%).

### Patients with ≥50% reduction (seizure responders) in seizure frequency

Twenty-nine studies reported 50% responder rates. Mean values and 95% confidence intervals were used as the aggregate data in the network meta-analysis, and the results revealed that compared with the placebo group, the seizure frequency reduction ≥50% are BRV 2.28 (95% CI 2.00 to 2.60), CNB 3.42 (95% CI 2.34 to 5.01), ESL 2.12 (95% CI 1.61 to 2.79), LCM 1.88 (95% CI 1.53 to 2.30), and PER 2.01 (95% CI 1.83 to 2.22). Therefore, the 50% responder rate of the CNB group was the highest, and that of the LCM group was the lowest. However, the 50% responder rate for the five drugs were significantly higher than that of placebo (Figures 4B, 5B).

Figure 6B shows the seizure response reduction of all treatments ranked by SUCRA probabilities. SUCRA analysis revealed that CNB achieved the best effect on the proportion of patients with ≥50% reduction in seizure frequency (SUCRA 98.52%), followed by BRV (SUCRA 72.08%).

### Rates of treatment-emergent adverse events

We analyzed the incidence of adverse events after treatment with BRV, CNB, ESL, LCM, PER, and placebo. The mean value in the LCM group was 2.04 (95% CI 1.43 to 2.91), as shown in Figures 4C, 5C, indicating the highest rate of TEAEs. The PER group 1.67 (95% CI 1.43 to 1.95) and the ESL group 1.87 (95% CI 1.53 to 2.30) also produced higher rates of adverse reactions. The mean value in the BRV group was 1.26 (95% CI 1.06 to 1.49), indicating the lowest rate of TEAEs. Based on these two outcome measures, ESL and CNB were ranked first. The rate of TEAEs was higher in ESL than in CNB. Figure 6C shows the rates of TEAE ranked according to SUCRA probabilities. SUCRA analysis revealed that LCM had the highest rate of TEAEs (SUCRA 12.36%), followed by ESL (SUCRA 19.6%).

### Evaluation of publication bias

**Figure 7:**
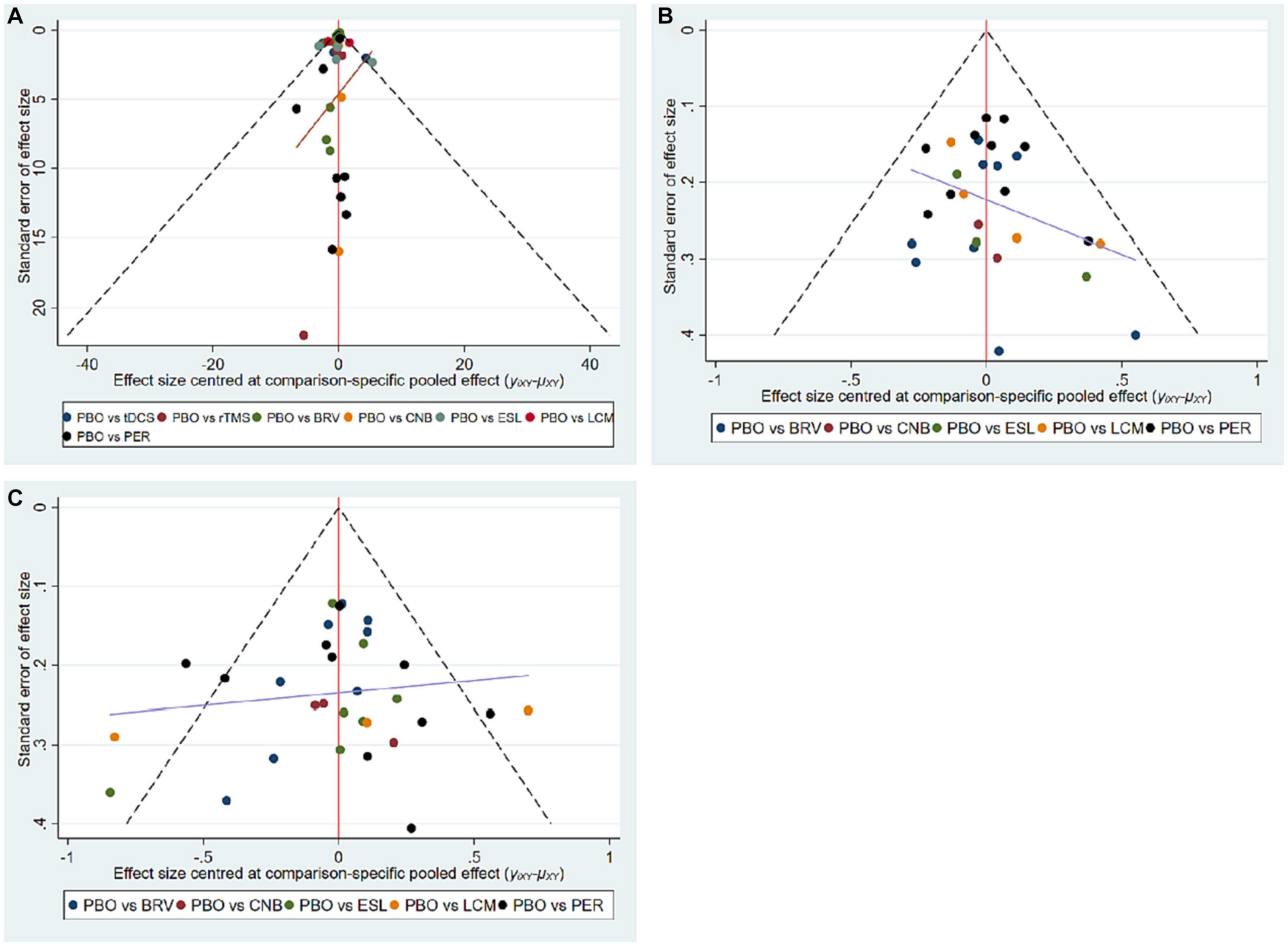
Funnel plots of multiple interventions for seizure frequency reduction from baseline **(A)**, 50% responder rate **(B)**, and TEAE **(C)**. TEAE, treatment-emergent adverse event; PBO, placebo; BRV, brivaracetam; CNB, cenobamate; ESL, eslicarbazepine acetate; LCM, lacosamide; PER, perampanel; rTMS, repetitive transcranial magnetic stimulation; tDCS, transcranial direct current stimulation.

We assessed publication bias in 32 studies that included seizure frequency (Figure 7A), 29 studies that included a 50% responder rate (Figure 7B), and 32 studies that included TEAEs (Figure 7C). The funnel plots reveal that the scatter was almost symmetrical, suggesting that the included trials had a relatively low publication bias.

## Discussion

Epilepsy is a prevalent and intricate disorder of the central nervous system resulting from the extensively synchronized discharge of neurons. About 30%–40% of patients with epilepsy are still unable to effectively control their seizures under the treatment of appropriate ASMs, which is defined as refractory epilepsy. The etiology of intractable epilepsy is multifaceted, encompassing diverse factors associated with the environment, genetics, and medication (7, 26, 27). Pharmacotherapy plays a crucial role in mitigating the severity of epilepsy and enhancing the quality of life for patients (28).

This network meta-analysis updates the currently available correlational studies. We included high-quality RCTs and other cohort studies. Using a Bayesian approach, we conducted a network meta-analysis to comprehensively assess the effectiveness and safety of both drug and non-drug therapies in the treatment of epilepsy. SUCRA was used for ranking. The top three interventions for reducing seizure frequency from baseline were drug therapies. Both tDCS and rTMS were less effective in controlling seizures than the five third-generation ASMs. In terms of the 50% response rate, CNB, BRV, and ESL were ranked highest. Meanwhile, BRV, CNB, and PER were well tolerated; the safety of LCM was lowest among the five third-generation ASMs.

Our findings reveal that ESL and LCM, when used as add-on treatments, could effectively reduce seizure frequency. LCM and ESL are both known to block voltage-gated sodium channels (29). LCM and ESL exert selective effects on pathological currents induced by slow channels, thereby inhibiting the activation of synaptic currents. Therefore, the pathological current can be prevented from spreading and the neural network can be stabilized (29–31). The adverse event profile of LCM presented in this meta-analysis indicates that adverse events are common with LCM therapy, with blurred vision, coordination problems, dizziness, drowsiness, nausea, and vomiting being the most commonly reported adverse effects (32–34). Rosenow et al. (35) included 1,308 patients who were randomly assigned to receive LCM treatment. All patients reported at least one TEAE, but most TEAEs were mild or moderate in intensity. Therapeutic doses of 200–400 mg/day were well tolerated, however severe TEAEs were observed when 600 mg/day was used. During the clinical treatment, the therapeutic dose can be adjusted in a timely manner to the drug responsiveness of the individual patient, which is different from fixed-dose therapy in clinical trials. Therefore, adverse events associated with LCM may be more effectively managed and exhibit a lower occurrence rate in real-world clinical settings. It is well known that ASMs may impair sleep. Liguori et al. (36) summarized the effects of ASMs on sleep in patients with epilepsy and found that LCM was associated with the occurrence of daytime sleepiness. Interestingly, no significant adverse events were observed after short-term use of LCM, suggesting that the adverse effects appear to be related to the duration of the course of treatment (37). ESL shares structural similarities with carbamazepine and oxcarbazepine, while exhibiting a lower propensity for drug interactions (38). The most frequently observed TEAEs included dizziness, drowsiness, headache, and nausea, while serious TEAEs were reported in less than 1% of patients. Adjuvant therapy for ESL is usually well tolerated, with most adverse events being mild to moderate in severity (39–41). We included a total of 7 clinical studies on adjuvant ESL therapy, among which Krauss. et al. (42) included 1,447 patients (ESL: 1021 and placebo: 426) to analyze the TEAE during various doses of 400, 800, or 1,200 mg QD for ESL therapy. The results showed that starting with a lower dose of ESL had a lower incidence of TEAE. In addition, discontinuation due to TEAE was more frequent in patients receiving a maintenance dose of 1,200 mg QD. The tolerance of ESL could be improved by reducing the dose or the titration rate. Because the large sample study has the characteristics of large scale, representativeness, repeatability and high stability, we are more confident in judging the authenticity and importance of the result effect.

For the 50% responder rate, the CNB group showed the highest efficacy. CNB can inhibit voltage-gated sodium currents to decrease the excitation current and target γ-amino butyric acid receptors to enhance inhibitory currents (43–45). The extensive preclinical activity of CNB can be attributed to its dual pharmacodynamic activity, which modulates both excitatory and inhibitory neurotransmission (46). CNB, as a novel pharmacological agent, shows great potential in treating patients with refractory seizures. At the same time, CNB was well tolerated. Studies have reported that the incidence of CNB-associated TEAEs tends to decrease with continued treatment (47, 48). Privitera et al. (49) analyzed the efficacy and safety of CNB and seven other AEDs for the treatment of uncontrolled focal seizures. The results revealed that CNB was more likely to provide ≥50% seizure reduction, without increasing treatment discontinuation due to TEAEs. The dose-related central nervous system adverse effects associated with CNB were primarily drowsiness, dizziness, double vision, and gait and coordination disorders (44). However, studies have found that the incidence of adverse events is higher in patients who use CNB in conjunction with sodium channel blockers (50). The utilization of CNB as an adjunctive therapy raises concerns regarding potential drug interactions. Overall, these findings offer compelling evidence supporting the efficacy of CNB in reducing seizures and its favorable side effect profile, aligning with the outcomes of the current meta-analysis.

In the present study, BRV considerably affected the 50% responder rate and was ranked second to CNB. BRV acts as a potent ligand for synaptic vesicle protein 2A, exerting inhibitory effects on voltage-dependent sodium channels in neurons (51). BRV has a similar chemical structure to levetiracetam (LEV), with a broader antiepileptic spectrum and higher efficacy than LEV. Brandt et al. (52) conducted a comprehensive analysis of safety data related to BRV as an adjunct therapy for the treatment of focal seizures. The results showed that the incidence of TEAE during treatment in the BRV group was not significantly different from that in the placebo group. Additionally, our meta-analysis demonstrated that BRV exhibited the highest tolerability among the drugs assessed, with the lowest incidence of TEAEs, corroborating previous research findings (53, 54).

PER, a noncompetitive α-amino-3-hydroxy-5-methyl-4-isoxazolepropionic acid (AMPA) receptor antagonist, has been considered as a first-in-class ASM. PER reduces neuronal excitability by inhibiting the AMPA receptor-mediated synaptic transmission (55). In this network meta-analysis, PER showed moderate efficacy in reducing seizure frequency. Further, the TEAE rate was low. Dizziness was the most common adverse reaction and may exhibit a dose-dependent response. In addition, PER may increase the incidence of somnolence, fatigue, and irritability (56).

At present, a wide range of studies have confirmed the efficacy and safety of NIBS in central nervous system diseases such as depression (57), Parkinson’s disease (58), stroke (59), Alzheimer’s Disease (60) and so on. Owing to its effects on modulating neuronal excitability and its high tolerability, NIBS has garnered increasing attention for the treatment of epilepsy (18). Six studies were analyzed which included 206 patients (tDCS: 79, rTMS: 47 and placebo: 80). Most patients had a slight itching sensation at the beginning of tDCS stimulation, and this discomfort disappeared immediately after the stimulation ended. In few patients, headache after tDCS treatment is of short duration and can be resolved on its own (61). Yang et al. (61) and San-Juan et al. (16) reported that even patients with refractory epilepsy who had a history of craniocerebral injury or surgery were able to tolerate tDCS intervention well. Rezakhani et al. (62) demonstrated that the quality of life of patients with refractory epilepsy improved significantly after 3 months of tDCS intervention compared with sham group, and that tDCS can improved cognition as well. We included a total of 3 studies on rTMS (rTMS: 47 and placebo: 41). A randomized, sham-controlled study used cognitive assessment as a secondary outcome measure for rTMS intervention to initially evaluate the safety of rTMS. The results showed that rTMS intervention group improved working memory, reactivity, attention and so on (63). Cantello et al. (64) evaluated the efficacy of rTMS in controlling seizures by seizure frequency and EEG changes. In this study, no significant or persistent side effects were reported, and medical and neurological examinations were unchanged. The included studies in our network meta-analysis study verified the safety of NIBS through some subjective scales and objective tests, but more high-quality clinical studies with larger sample size and more objective indicators are needed for further research in the future.

Transcranial magnetic stimulation (TMS) is a well-tolerated technique that effectively stimulates both excitatory and inhibitory neurons within the cerebral cortex without causing discomfort (65). rTMS has been widely shown to induce long-lasting effects after consecutive sessions (66, 67). When an electric current passes through the coil, it generates a magnetic field that has the potential to induce a localized intracranial electric current within the brain, effectively reaching and stimulating the desired brain tissue (68). rTMS produces long-term inhibitory effects on synaptic potentials and focal cortical excitability, which may reduce the rate of seizures (69). The application of rTMS in the treatment of central nervous system diseases holds promise, but it is important to acknowledge its limitations. It is worth noting that rTMS can induce seizures when the frequency is high and the stimulation interval is short (70). The bidirectional regulation of human cortical excitability can be achieved by adjusting the stimulation rate (71). Further studies focusing on personalized rTMS parameters may be required to maximize the therapeutic outcomes of this technique for brain stimulation in clinical settings. A small number of people had mild dizziness or headaches during treatment, and no significant or persistent side effects were reported.

tDCS, through the application of direct currents on the intact scalp, has the ability to induce enduring changes in cortical excitability in the human brain. The stimulation is released by placing a relatively large area of electrodes on the scalp area of interest (72). The flow of current through the targeted neuronal tissue in a specific direction leads to a polarity-dependent alteration in the resting membrane potential (73). Like rTMS, tDCS can modulate neuronal excitability in both directions as well. The effect of cathode tDCS is similar to that of low frequency rTMS, which is conducive to enhancing inhibition (74). Compared to adult patients, inpatient children appear to have higher 50% responder rates after tDCS treatment (75, 76). However, larger studies are required to confirm whether younger patients should preferentially receive tDCS as a treatment for epilepsy. No serious adverse events related to the application of tDCS have been reported. Minor local skin itching and tingling are common TEAEs, and these adverse reactions can be cured by themselves (77).

Patients with epilepsy in special situations, such as pregnant individuals, require tailored considerations during their treatment due to the unique challenges posed by their condition. The high safety profile of NIBS therapy, a non-surgical and non-drug treatment, renders it potentially significant for addressing the unique needs of epilepsy patients in special populations. During pregnancy, the pharmacokinetics of ASMs change. These changes may potentially affect seizure frequency and fetal exposure to ASMs, and even carry the risk of teratogenic effects (78, 79). Laurin et al. (80) presented three case reports demonstrating that tDCS appears to be a safe and effective treatment for many mental disorders in the perinatal period, including depression and post-traumatic stress disorder. Pregnant patients with treatment-resistant depression exhibited favorable tolerability to rTMS, with more than 50% of patients in the intervention group showing improved mood after the treatment period ended (81). Multiple clinical studies and high-quality systematic reviews have provided evidence regarding the efficacy and safety of NIBS in pregnant patients with depression (82–84). rTMS and tDCS, which can influence synaptic transmission to alter neuronal excitability (82, 83), appear to be a potential additional approach for patients with epilepsy during pregnancy to reduce the use of drugs and thus reduce drug-related risks. However, there is currently a lack of studies investigating the use of NIBS in pregnant patients with epilepsy, and more evidence is needed to verify the feasibility of this hypothesis in the future.

In summary, both rTMS and tDCS show great potential as therapeutic approaches for individuals with epilepsy. Nevertheless, the clinical advantages of these techniques should be validated through larger-scale, double-blind, randomized trials.

This study has some limitations. The RCTs and other cohort studies included were placebo-controlled; hence direct comparison of the different treatments was not possible. The network meta-analysis lacked adequate dose limitations in the included trials, which may have limited the comprehensive evaluation of therapeutic effects for the interventions. Hence, additional studies are necessary to address these limitations and facilitate the derivation of more precise and specific conclusions.

## Conclusion

This study revealed that CNB, ESL, and LCM are more effective in controlling seizures, among the five third-generation antiseizure medications. BRV exhibited the lowest occurrence rate of adverse events. Moreover, rTMS and tDCS exhibit satisfactory safety profiles. In the future, it is necessary to conduct high-quality randomized controlled trials and other cohort studies to validate the findings of this study.

## Data availability statement

The original contributions presented in the study are included in the article/Supplementary material, further inquiries can be directed to the corresponding author.

## Author contributions

YY: Writing – original draft, Writing – review & editing. YFS: Writing – original draft, Writing – review & editing. XMW: Data curation, Funding acquisition, Investigation, Writing – review & editing. RHL: Formal analysis, Methodology, Software, Writing – review & editing. ZYS: Data curation, Investigation, Writing – review & editing. MT: Formal analysis, Methodology, Software, Writing – review & editing. GHJ: Funding acquisition, Project administration, Supervision, Writing – review & editing.
