# Supplementary material for: The efficacy and safety of third-generation antiseizure medications and non-invasive brain stimulation to treat refractory epilepsy: a systematic review and network meta-analysis study
Source: Front Neurol. 2024 Jan 9;14:1307296. doi: 10.3389/fneur.2023.1307296 (PMC10804851; doi:10.3389/fneur.2023.1307296)
Supplement: Supplementary file 1 [file Table_1.DOCX]

| **Id** | **Study** | **Year** | **Design** | **Sample size** | **Treatment group inventions** | **Control group inventions** | **Outcome measures** | **NOS scores** |
| --- | --- | --- | --- | --- | --- | --- | --- | --- |
| 1 | San-Juan et al.[1] | 2016 | Randomized, double-blinded, placebo-controlled, 3-arm parallel group clinical trial | tDCS 20, placebo 8 | tDCS:30 min × 3d,  30 min × 5d | Sham stimulation | Primary: a decrease in seizure frequency compared to the baseline.  Secondary: Epileptiform Discharges in EEGs; mean reduction of seizure monthly frequency; safety and adverse events. | - |
| 2 | Yang et al.[2] | 2019 | Randomized, double-blind, sham-controlled, three-arm parallel group multicenter study | tDCS 49, placebo 21 | tDCS: 20-minutes active stimulation,  2 × 20-minutes active stimulation | Sham stimulation | Primary: seizure frequency.  Secondary: the Quality of Life in Epilepsy-31 Inventory, the number of seizures occurred during the tDCS stimulation period; safety and adverse events. | - |
| 3 | Rezakhani et al.[3] | 2022 | Randomized placebo-controlled and double-blind clinical trial | tDCS 10, placebo 10 | tDCS: 30-minutes active stimulation | Sham stimulation | Primary: Seizure frequency and IEDs frequency.  Secondary: Quality of Life in Epilepsy Inventory and Cognitive Assessment scores. | - |
| 4 | Fregni et al.[4] | 2006 | Randomized, double-blind, sham-controlled, parallel design clinical trial | rTMS 12, placebo 9 | rTMS: stimulation frequency of 1Hz | Sham stimulation | Primary: Seizure frequency.  Secondary: Epileptiform discharges, cognitive assessment. | - |
| 5 | Theodore et al.[5] | 2002 | Controlled study | rTMS 12, placebo 12 | rTMS: stimulation frequency of 1Hz | Sham stimulation | Seizure frequency. | 7 |
| 6 | Cantello et al.[6] | 2007 | Randomized, double-blind, sham-controlled, crossover study | rTMS 23, placebo 20 | rTMS: stimulation frequency of 0.3Hz | Sham stimulation | Primary: Seizure frequency.  Secondary: EEG epileptiform abnormalities, resting. motor threshold, safety and adverse events. | - |
| 7 | Ben-Menachem et al.[7] | 2016 | Randomized, double-blind, placebo-controlled study | BRV 742, placebo 418 | BRV: 50,100,200 mg/day | Placebo | Primary: percent reduction in POS frequency over placebo per 28 days and 50% responder rate.  Secondary: percent reduction in POS frequency from baseline to treatment period, seizure freedom during the treatment period, and categorized reduction in POS frequency from baseline to treatment period, safety and adverse events. | 7 |
| 8 | Lee et al.[8] | 2021 | Randomized, double-blind, placebo-controlled trial | BRV 742, placebo 418 | BRV: 50,100,200 mg/day | Placebo | 50% responder rate (50% reduction in focal seizure frequency from baseline), median percentage reduction in 28-day adjusted focal seizure frequency from baseline, percentage reduction over placebo in 28-day adjusted focal seizure frequency, and seizure freedom (including all seizure types) during the treatment period, safety and adverse events. | - |
| 9 | Klein et al.[9] | 2020 | Randomized, double-blind, placebo (PBO)-controlled Phase III trial | BRV 503, placebo 261 | BRV: 100,200 mg/day | Placebo | 50%, 75% and 100% responder rates (patients with a ≥50%, ≥75% or 100% reduction from Baseline in focal seizure frequency/28 days), safety and adverse events. | - |
| 10 | French et al.[10] | 2010 | Randomized, double-blind, placebo-controlled, international, parallel-group dose-ranging study | BRV 154, placebo 54 | BRV: 5,20,50 mg/day | Placebo | Primary: reduction in baseline-adjusted weekly POS frequency compared to placebo.  Secondary: percentage reduction from baseline in weekly POS frequency; 50% responder rate for POS; freedom from POS; safety and adverse events. | 8 |
| 11 | Paesschen et al.[11] | 2013 | Randomized, double-blind, placebo-controlled, multicenter, parallel group, dose-ranging study | BRV 100, placebo 48 | BRV: 50,100 mg/day | Placebo | Primary: the percent reduction in baseline-adjusted partial-onset seizure frequency/week over placebo. Secondary: the reduction in partial-onset seizure frequency/week over placebo; percent reduction from baseline in partial-onset seizure frequency/week; 50%responder rate; and freedom from partial-onset seizures; safety and adverse events. | 7 |
| 12 | Kwan et al.[12] | 2014 | A phase III, double-blind, randomized, placebo-controlled, flexible-dose trial | BRV 323, placebo 111 | BRV: 20-150 mg/day | Placebo | Primary: safety and adverse events.  Secondary: the percent reduction in baselineadjusted focal seizure frequency/week; the median percent reduction from baseline in focal seizure frequency/week; ≥50% responder rate in focal seizure frequency/week, seizure freedom rate, and time to nth focal seizure | 7 |
| 13 | Klein et al.[13] | 2015 | Randomized, double-blind, placebo-controlled, multicenter, parallel-group study | BRV 450, placebo 246 | BRV: 100,200 mg/day | Placebo | Primary: percent reduction over PBO in 28-day adjusted POS frequency, and ≥50% responder rate based on percent reduction in seizure frequency from baseline.  Secondary: percent reduction in seizure frequency from baseline to the treatment period, categorized percent reduction from baseline in seizure frequency over the treatment period, and seizure freedom rate; safety and adverse events | - |
| 14 | Biton et al.[14] | 2014 | A phase III randomized, double-blind, placebo-controlled trial | BRV 296, placebo 96 | BRV: 5,20,50 mg/day | Placebo | Primary: the partial-onset seizure frequency/week, with the percent reduction over PBO. Secondary: median percent reduction from baseline in partial-onset seizure frequency/week, ≥50% responder rate, and seizure freedom from all seizure types; safety and adverse events. | 6 |
| 15 | Ryvlin et al.[15] | 2014 | A double-blind, randomized, placebo-controlled trial | BRV 298, placebo 100 | BRV: 20,50,100 mg/day | Placebo | Primary: the focal seizure frequency/week. Secondary: the median percent reduction from baseline in focal seizure frequency/week; ≥50% responder rate; seizure freedom from all seizure types; safety and adverse events. | 5 |
| 16 | Klein et al.[16] | 2016 | Randomized phase III studies | BRV 742, placebo 418 | BRV: 50,100,200 mg/day | Placebo | Time to onset of sustained ≥50% responder status. | 8 |
| 17 | Krauss et al.[17] | 2019 | A multinational, multicentre, double-blind, randomised, placebo-controlled, dose-response study | CNB 266, placebo 94 | CNB: 100,200,400 mg/day | Placebo | Primary: the percentage change from baseline in focal seizure frequency averaged over 28 days; the responder rate . Secondary: the percentage change in seizure frequency; the responder rate. | - |
| 18 | Chung et al.[18] | 2020 | Multicenter, double-blind, placebo-controlled study | CNB 113, placebo 108 | CNB: 200 mg/day | Placebo | Primary: percent change from baseline in focal seizure frequency per 28 days. Secondary: responder rate; assessment of seizure frequency by seizure type; safety and adverse events. | 7 |
| 19 | Rosenfeld et al.[19] | 2021 | Multicenter, multinational, double-blind, randomized, placebo-controlled study | CNB 295, placebo 102 | CNB: 100,200,400 mg/day | Placebo | median percent reduction in seizure frequency/28 days; responder rates from baseline; safety and adverse events. | - |
| 20 | Elger et al.[20] | 2009 | Randomized, double-blind, placebo-controlled, parallel-group phase III study | ESL 246, placebo 84 | ESL: 400,800,1200 mg/day | Placebo | Primary: the change in seizure frequency compared to baseline. Secondary: responder rates; safety and adverse events. | 8 |
| 21 | Ben-Menachem et al.[21] | 2010 | Double-blind, randomized, placebo-controlled, parallel-group, Phase III study | ESL 295, placebo 100 | ESL: 400,800,1200 mg/day | Placebo | Primary: the change in seizure frequency compared to baseline. Secondary: responder rates; safety and adverse events. | - |
| 22 | Elger et al.[22] | 2007 | A multicenter, randomized, placebo-controlled, parallel-group, therapeutic exploratory (phase II) study | ESL 96, placebo 47 | ESL: 400,800,1200 mg/day,qd 200,400,800 mg/day,bid | Placebo | Primary: responder rates. Secondary: reduction in seizure frequency compared to the baseline; safety and adverse events. | - |
| 23 | Sperling et al.[23] | 2014 | A phase III, double-blind, randomized, placebo-controlled trial | ESL 426, placebo 224 | ESL: 800,1200 mg/day | Placebo | Primary: seizure frequency. Secondary: responder rate; reduction in seizure frequency; seizure freedom; CGI and QOLIE-31 scores; safety and adverse events. | 7 |
| 24 | Krauss et al.[24] | 2018 | An integrated analysis of three randomized placebo-controlled trials | ESL 1021, placebo 426 | ESL: 400,800 mg/day | Placebo | safety and adverse events. | 8 |
| 25 | Gil-Nagel et al.[25] | 2009 | Double-blind, randomized, placebo-controlled, parallel- group, Phase III study | ESL 129, placebo 65 | ESL: 800,1200 mg/day | Placebo | Primary: seizure frequency standardized per 4 weeks. Secondary: the proportion of responders, relative reduction in seizure frequency, number of days with seizures, distribution of seizure reduction, proportion of seizure-free patients, proportion of patients with an exacerbation in seizure frequency ≥25% compared to baseline; safety and adverse events. | 6 |
| 26 | Kirkham et al.[26] | 2020 | Double-blind, randomized, placebo-controlled, parallel-group, multicenter, phase-III clinical trial | ESL 134, placebo 129 | ESL: 10-30 mg/day | Placebo | Primary: responder rate, the relative reduction in seizure frequency. Secondary: safety and adverse events. | 6 |
| 27 | Halasz et al.[27] | 2009 | Multicenter, double-blind, randomized, placebo-controlled, parallel-group trial | LCM 258, placebo 141 | LCM:200,400 mg/day | Placebo | Primary: the change in seizure frequency per 28 days from baseline; the 50% responder rate. Secondary: the percent change in seizure frequency per 28 days from baseline, number and proportion of patients achieving seizure-free status, proportion of seizure-free days during the maintenance period for patients entering the maintenance period. | - |
| 28 | Ben-Menachem et al.[28] | 2007 | Multicenter, multinational, double-blind, placebo-controlled, randomized, dose-response trial | LCM 319, placebo 96 | LCM:200,400,600 mg/day | Placebo | Primary: reduction in seizure frequency per 28 days from baseline, responder rate. Secondary: percent change in seizure frequency, achievement of seizure-free status, proportion of seizure-free days, Clinical Global Impression of Change (CGIC) score, Quality of Life in Epilepsy (QOLIE-31) questionnaire score; safety and adverse events. | - |
| 29 | Chung et al.[29] | 2010 | Randomized, double-blind, placebo-controlled, parallel-group trial | LCM 298, placebo 104 | LCM:400,600 mg/day | Placebo | Primary: the change in seizure frequency per 28 days from baseline, 50% responder rate. Secondary: the percent change in seizure frequency per 28 days from baseline; the 75% responder rate; the number and proportion of patients achieving seizure-free status; the percentage of seizure-free days throughout maintenance for patients who entered maintenance; the change in seizure frequency and 50% responder rate differentiated by seizure type; safety and adverse events. | - |
| 30 | Biton et al.[30] | 2015 | Randomized, double-blind, placebo-controlled trials | LCM 944, placebo 364 | LCM:200,400,600 mg/day | Placebo | safety and adverse events. | - |
| 31 | Sperling et al.[31] | 2013 | Randomized, double-blind, placebo-controlled trial | LCM 935, placebo 359 | LCM:200,400,600 mg/day | Placebo | change in partial seizure frequency per 28 days from baseline; 50% responder rate. | - |
| 32 | Inoue et al.[32] | 2021 | Phase III, uncontrolled, flexible-dose, open-label, multicenter extension trial | LCM 309, placebo 164 | LCM:200,400 mg/day | Placebo | Primary: percent change in focal seizure frequency per 28 days from the Baseline; ≥50 % responder rate. Secondary: ≥75 % responder rate; percentage of seizure-free days; safety and adverse events. | - |
| 33 | Tsai et al.[33] | 2017 | Phase‐3 multicenter, randomized, double‐blind, placebo‐controlled studies | PER 1569, placebo 618 | PER:2,4,8,12 mg/day | Placebo | Primary: percent change in seizure frequency per 28 days relative to baseline; 50%, 75%, and 100% responder rates. Secondary: safety and adverse events. | - |
| 34 | Steinhoff et al.[34] | 2019 | Randomized, double-blind, phase III studies | PER 365, placebo 618 | PER:4 mg/day | Placebo | median percentage reductions in seizure frequency per 28 days from baseline; 50% and 75% responder rates; seizure-freedom rates; safety and adverse events. | 7 |
| 35 | Nishida et al.[35] | 2017 | Randomized, double-blind, placebo-controlled, parallel-group study | PER 531, placebo 176 | PER:4,8,12 mg/day | Placebo | Primary: percent change in POS frequency per 28 days. Secondary: 50% responder rate, seizure-freedom rate, safety and adverse events. | 5 |
| 36 | French et al.[36] | 2012 | Multicenter, multinational, randomized, double-blind, placebo-controlled trial | PER 267, placebo 121 | PER:8,12 mg/day | Placebo | Median percentage change; responder rate; safety and adverse events. | 7 |
| 37 | Nishida et al.[37] | 2018 | post hoc analysis | PER 1650, placebo 700 | PER:2,4,8,12 mg/day | Placebo | Percentage change in focal seizures with or without FBTC or GTC seizure frequency per 28 days relative to baseline; 50% responder rate; safety and adverse events. | 8 |
| 38 | Meador et al.[38] | 2016 | Randomized, double-blind, placebo-controlled, parallel-group phase II study | PER 85, placebo 48 | PER:8-12 mg/day | Placebo | Safety and adverse events. | 8 |
| 39 | Montouris et al.[39] | 2015 | Multinational, double-blind, placebo-controlled, phase III core studies | PER 838, placebo 378 | PER:2,4,8,12 mg/day | Placebo | Primary: the median percent reduction in total seizure frequency per 28 days of treatment relative to baseline; 50% responder rate. Secondary: percent change frequency per 28 days during treatment relative to baseline; safety and adverse events. | 6 |
| 40 | Krauss et al.[40] | 2012 | Multicenter, multinational, double-blind, placebo-controlled, randomized, dose-response trial | PER 521, placebo 185 | PER:2,4,8 mg/day | Placebo | Primary: the percent change in seizure frequency per 28 days; 50% responder rate. Secondary: the percent change in the frequency of complex partial seizures plus secondarily generalized seizures; a dose-response analysis of the percent change in seizure frequency; safety and adverse events. | 8 |
| 41 | Steinhoff et al.[41] | 2013 | A pooled analysis of three phase III studies | PER 1038, placebo 442 | PER:2,4,8,12 mg/day | Placebo | Primary: median percentage change in the frequency of all partial seizures per 28 days; 50% responder rate. Secondary: 75% responder rates; seizurefreedom rates; safety and adverse events. | 5 |
| 42 | Liao et al.[42] | 2020 | Post hoc analysis of phase III double-blind and open-label extension studies | PER 198, placebo 79 | PER:2,4,8,12 mg/day | Placebo | Median percent change in seizure frequency per 28 days relative to baseline; 50% and 75% responder rates; seizure-freedom rates; safety and adverse events. | 7 |
| 43 | French et al.[43] | 2013 | Multicenter, multinational, randomized, double-blind, placebo-controlled trial | PER 250, placebo 136 | PER:8,12 mg/day | Placebo | 50% responder rate; median percentage change in seizure frequency per 28 days compared with baseline. | 6 |
| 44 | Gidal et al.[44] | 2013 | Multicenter, randomized, double-blind, placebo-controlled, parallel-group phase III studies | PER 770, placebo 339 | PER:2,4,8,12 mg/day | Placebo | Primary: median percent change from baseline in overall seizure frequency per 28 days; 50% responder rate. | 8 |
| 45 | Ko et al.[45] | 2015 | Multinational, multicenter, randomized, double-blind, and PBO-controlled. | PER 1038, placebo 442 | PER:2,4,8,12 mg/day | Placebo | Safety and adverse events. | 6 |

**Supplementary Table 1: Summary of clinical trials included in the network meta-analysis. PBO: placebo; BRV: Brivaracetam; CNB: cenobamate; ESL: eslicarbazepine acetate; LCM: lacosamide; PER: perampanel, rTMS: repetitive transcranial magnetic stimulation; tDCS: transcranial direct current stimulation.**

1. San-Juan, D., et al., *Transcranial Direct Current Stimulation in Mesial Temporal Lobe Epilepsy and Hippocampal Sclerosis.* Brain Stimul, 2017. **10**(1): p. 28-35.

2. Yang, D., et al., *Transcranial direct current stimulation reduces seizure frequency in patients with refractory focal epilepsy: A randomized, double-blind, sham-controlled, and three-arm parallel multicenter study.* Brain Stimulation, 2020. **13**(1): p. 109-116.

3. Rezakhani, S., et al., *Therapeutic efficacy of seizure onset zone-targeting high-definition cathodal tDCS in patients with drug-resistant focal epilepsy.* Clinical Neurophysiology : Official Journal of the International Federation of Clinical Neurophysiology, 2022. **136**: p. 219-227.

4. Fregni, F., et al., *A randomized clinical trial of repetitive transcranial magnetic stimulation in patients with refractory epilepsy.* Ann Neurol, 2006. **60**(4): p. 447-55.

5. Theodore, W.H., et al., *Transcranial magnetic stimulation for the treatment of seizures: a controlled study.* Neurology, 2002. **59**(4): p. 560-562.

6. Cantello, R., et al., *Slow repetitive TMS for drug-resistant epilepsy: clinical and EEG findings of a placebo-controlled trial.* Epilepsia, 2007. **48**(2): p. 366-74.

7. Ben-Menachem, E., et al., *Efficacy and safety of brivaracetam for partial-onset seizures in 3 pooled clinical studies.* Neurology, 2016. **87**(3): p. 314-323.

8. Lee, S.K., et al., *Effect of Number of Previous Antiseizure Medications on Efficacy and Tolerability of Adjunctive Brivaracetam for Uncontrolled Focal Seizures: Post Hoc Analysis.* Adv Ther, 2021. **38**(7): p. 4082-4099.

9. Klein, P., et al., *Effect of lifetime antiepileptic drug treatment history on efficacy and tolerability of adjunctive brivaracetam in adults with focal seizures: Post-hoc analysis of a randomized, placebo-controlled trial.* Epilepsy Res, 2020. **167**: p. 106369.

10. French, J.A., et al., *Adjunctive brivaracetam for refractory partial-onset seizures: a randomized, controlled trial.* Neurology, 2010. **75**(6): p. 519-525.

11. Van Paesschen, W., et al., *Efficacy and tolerability of adjunctive brivaracetam in adults with uncontrolled partial-onset seizures: a phase IIb, randomized, controlled trial.* Epilepsia, 2013. **54**(1): p. 89-97.

12. Kwan, P., et al., *Adjunctive brivaracetam for uncontrolled focal and generalized epilepsies: results of a phase III, double-blind, randomized, placebo-controlled, flexible-dose trial.* Epilepsia, 2014. **55**(1): p. 38-46.

13. Klein, P., et al., *A randomized, double-blind, placebo-controlled, multicenter, parallel-group study to evaluate the efficacy and safety of adjunctive brivaracetam in adult patients with uncontrolled partial-onset seizures.* Epilepsia, 2015. **56**(12): p. 1890-8.

14. Biton, V., et al., *Brivaracetam as adjunctive treatment for uncontrolled partial epilepsy in adults: a phase III randomized, double-blind, placebo-controlled trial.* Epilepsia, 2014. **55**(1): p. 57-66.

15. Ryvlin, P., et al., *Adjunctive brivaracetam in adults with uncontrolled focal epilepsy: results from a double-blind, randomized, placebo-controlled trial.* Epilepsia, 2014. **55**(1): p. 47-56.

16. Klein, P., et al., *Time to onset of sustained >/=50% responder status in patients with focal (partial-onset) seizures in three phase III studies of adjunctive brivaracetam treatment.* Epilepsia, 2017. **58**(2): p. e21-e25.

17. Krauss, G.L., et al., *Safety and efficacy of adjunctive cenobamate (YKP3089) in patients with uncontrolled focal seizures: a multicentre, double-blind, randomised, placebo-controlled, dose-response trial.* Lancet Neurol, 2020. **19**(1): p. 38-48.

18. Chung, S.S., et al., *Randomized phase 2 study of adjunctive cenobamate in patients with uncontrolled focal seizures.* Neurology, 2020. **94**(22): p. e2311-e2322.

19. Rosenfeld, W.E., A. Nisman, and L. Ferrari, *Efficacy of adjunctive cenobamate based on number of concomitant antiseizure medications, seizure frequency, and epilepsy duration at baseline: A post-hoc analysis of a randomized clinical study.* Epilepsy Res, 2021. **172**: p. 106592.

20. Elger, C., et al., *Efficacy and safety of eslicarbazepine acetate as adjunctive treatment in adults with refractory partial-onset seizures: a randomized, double-blind, placebo-controlled, parallel-group phase III study.* Epilepsia, 2009. **50**(3): p. 454-63.

21. Ben-Menachem, E., et al., *Eslicarbazepine acetate as adjunctive therapy in adult patients with partial epilepsy.* Epilepsy Res, 2010. **89**(2-3): p. 278-85.

22. Elger, C., et al., *Eslicarbazepine acetate: a double-blind, add-on, placebo-controlled exploratory trial in adult patients with partial-onset seizures.* Epilepsia, 2007. **48**(3): p. 497-504.

23. Sperling, M.R., et al., *Eslicarbazepine acetate as adjunctive therapy in patients with uncontrolled partial-onset seizures: Results of a phase III, double-blind, randomized, placebo-controlled trial.* Epilepsia, 2015. **56**(2): p. 244-53.

24. Krauss, G., et al., *Influence of titration schedule and maintenance dose on the tolerability of adjunctive eslicarbazepine acetate: An integrated analysis of three randomized placebo-controlled trials.* Epilepsy Res, 2018. **139**: p. 1-8.

25. Gil-Nagel, A., et al., *Efficacy and safety of 800 and 1200 mg eslicarbazepine acetate as adjunctive treatment in adults with refractory partial-onset seizures.* Acta Neurol Scand, 2009. **120**(5): p. 281-7.

26. Kirkham, F., et al., *Efficacy and safety of eslicarbazepine acetate as adjunctive therapy for refractory focal-onset seizures in children: A double-blind, randomized, placebo-controlled, parallel-group, multicenter, phase-III clinical trial.* Epilepsy Behav, 2020. **105**: p. 106962.

27. Halasz, P., et al., *Adjunctive lacosamide for partial-onset seizures: Efficacy and safety results from a randomized controlled trial.* Epilepsia, 2009. **50**(3): p. 443-53.

28. Ben-Menachem, E., et al., *Efficacy and safety of oral lacosamide as adjunctive therapy in adults with partial-onset seizures.* Epilepsia, 2007. **48**(7): p. 1308-17.

29. Chung, S., et al., *Lacosamide as adjunctive therapy for partial-onset seizures: a randomized controlled trial.* Epilepsia, 2010. **51**(6): p. 958-67.

30. Biton, V., et al., *Safety and tolerability of lacosamide as adjunctive therapy for adults with partial-onset seizures: Analysis of data pooled from three randomized, double-blind, placebo-controlled clinical trials.* Epilepsy Behav, 2015. **52**(Pt A): p. 119-27.

31. Sperling, M.R., et al., *Efficacy of lacosamide by focal seizure subtype.* Epilepsy Res, 2014. **108**(8): p. 1392-8.

32. Inoue, Y., et al., *Safety and efficacy of adjunctive lacosamide in Chinese and Japanese adults with epilepsy and focal seizures: A long-term, open-label extension of a randomized, controlled trial.* Epilepsy Res, 2021. **176**: p. 106705.

33. Tsai, J.-J., et al., *Efficacy, safety, and tolerability of perampanel in Asian and non-Asian patients with epilepsy.* Epilepsia, 2019. **60 Suppl 1**: p. 37-46.

34. Steinhoff, B.J., et al., *Efficacy and safety of adjunctive perampanel 4 mg/d for the treatment of focal seizures: A pooled post hoc analysis of four randomized, double-blind, phase III studies.* Epilepsia, 2020. **61**(2): p. 278-286.

35. Nishida, T., et al., *Adjunctive perampanel in partial-onset seizures: Asia-Pacific, randomized phase III study.* Acta Neurol Scand, 2018. **137**(4): p. 392-399.

36. French, J.A., et al., *Adjunctive perampanel for refractory partial-onset seizures: randomized phase III study 304.* Neurology, 2012. **79**(6): p. 589-596.

37. Nishida, T., et al., *Efficacy and safety of perampanel in generalized and focal to bilateral tonic-clonic seizures: A comparative study of Asian and non-Asian populations.* Epilepsia, 2019. **60 Suppl 1**: p. 47-59.

38. Meador, K.J., et al., *Cognitive effects of adjunctive perampanel for partial-onset seizures: A randomized trial.* Epilepsia, 2016. **57**(2): p. 243-51.

39. Montouris, G., et al., *Efficacy and safety of perampanel in patients with drug-resistant partial seizures after conversion from double-blind placebo to open-label perampanel.* Epilepsy Res, 2015. **114**: p. 131-40.

40. Krauss, G.L., et al., *Randomized phase III study 306: adjunctive perampanel for refractory partial-onset seizures.* Neurology, 2012. **78**(18): p. 1408-1415.

41. Steinhoff, B.J., et al., *Efficacy and safety of adjunctive perampanel for the treatment of refractory partial seizures: a pooled analysis of three phase III studies.* Epilepsia, 2013. **54**(8): p. 1481-9.

42. Weiping, L., et al., *Efficacy, safety, and tolerability of adjunctive perampanel in patients from China with focal seizures or generalized tonic-clonic seizures: Post hoc analysis of phase III double-blind and open-label extension studies.* CNS Neurosci Ther, 2021. **27**(3): p. 330-340.

43. French, J.A., et al., *Evaluation of adjunctive perampanel in patients with refractory partial-onset seizures: results of randomized global phase III study 305.* Epilepsia, 2013. **54**(1): p. 117-25.

44. Gidal, B.E., et al., *Concentration-effect relationships with perampanel in patients with pharmacoresistant partial-onset seizures.* Epilepsia, 2013. **54**(8): p. 1490-7.

45. Ko, D., et al., *Perampanel in the treatment of partial seizures: Time to onset and duration of most common adverse events from pooled Phase III and extension studies.* Epilepsy Behav, 2015. **48**: p. 45-52.
